# Spent Hen Protein Hydrolysate with Good Gastrointestinal Stability and Permeability in Caco-2 Cells Shows Antihypertensive Activity in SHR

**DOI:** 10.3390/foods9101384

**Published:** 2020-10-01

**Authors:** Hongbing Fan, Wenlin Yu, Wang Liao, Jianping Wu

**Affiliations:** 1 Department of Agricultural, Food and Nutritional Science, 4-10 Ag/For Building, University of Alberta, Edmonton, AB T6G 2P5, Canada; hongbing@ualberta.ca (H.F.); wenlin1@ualberta.ca (W.Y.); wliao@ualberta.ca (W.L.); 2 Cardiovascular Research Centre, University of Alberta, Edmonton, AB T6G 2R7, Canada

**Keywords:** spent hen, muscle proteins, ACE, ACE2, inflammation, oxidative stress, vascular cells, Caco-2, gastrointestinal digestion, spontaneously hypertensive rats

## Abstract

Spent hens are a major byproduct of the egg industry but are rich in muscle proteins that can be enzymatically transformed into bioactive peptides. The present study aimed to develop a spent hen muscle protein hydrolysate (SPH) with antihypertensive activity. Spent hen muscle proteins were hydrolyzed by nine enzymes, either individually or in combination; 18 SPHs were assessed initially for their in vitro angiotensin-converting enzyme (ACE) inhibitory activity, and three SPHs, prepared by Protex 26L (SPH-26L), pepsin (SPH-P), and thermoase (SPH-T), showed promising activity and peptide yield. These three hydrolysates were further assessed for their angiotensin-converting enzyme 2 (ACE2) upregulating, antioxidant, and anti-inflammatory activities; only SPH-T upregulated ACE2 expression, while all three SPHs showed antioxidant and anti-inflammatory activities. During simulated gastrointestinal digestion, ACE2 upregulating, ACE inhibitory and antioxidant activities of SPH-T were not affected, but those of SPH-26L and SPH-P were reduced. ACE inhibitory activity of gastrointestinal-digested SPH-T was not affected after the permeability study in Caco-2 cells, while ACE2 upregulating, antioxidant and anti-inflammatory activities were improved; nine novel peptides with five–eight amino acid residues were identified from the Caco-2 permeate. Among these three hydrolysates, only SPH-T reduced blood pressure significantly when given orally at a daily dose of 1000 mg/kg body weight to spontaneously hypertensive rats. SPH-T can be developed into a promising functional food ingredient against hypertension, contributing to a more sustainable utilization for spent hens while generating extra revenue for the egg industry.

## 1. Introduction

Hypertension is a global health concern and an important risk factor for cardiovascular diseases. Currently, pharmaceutical drugs such as angiotensin-converting enzyme (ACE) inhibitors and angiotensin (Ang) II type 1 receptor (AT_1_R) blockers are the first-line therapy for hypertension, but they are associated with various adverse effects such as dry cough and angioedema over a prolonged use [1]. Moreover, about one third of treated patients did not have their blood pressure adequately controlled [2,3]. Therefore, developing new alternatives such as natural compounds like antihypertensive peptides with a lower cost and fewer side effects has gained increasing interest over the past decades [4].

Numerous antihypertensive peptides have been characterized from various food proteins. Research has consistently shown a discrepancy between the in vivo efficacy of antihypertensive peptides and their in vitro activity; many hydrolysates or peptides possessing good in vitro activity failed to exert physiological effect in spontaneously hypertensive rats (SHRs), including hydrolysates produced from salmon, cod, and haddock, and peptides such as FKGRYYP (from chicken creatine kinase), FFGRCVSP (from egg ovalbumin), and ERKIKVYL (from egg ovalbumin), to name a few [5,6]. One possible reason is the presence of numerous proteases/peptidases in the gastrointestinal digestion and intestinal epithelium that may render peptides inactive. While being presented as major barriers, gastrointestinal digestion and transepithelial transport are also valuable strategies to assist generation of peptides with enhanced activities [5,7,8,9,10]. For example, two ovotransferrin-derived antihypertensive peptides IRW and IQW required pepsin to liberate from their respective parent peptides IRWCT and IQWCA [11]. A meat-derived peptide IWHHT liberated IWH and IW during gastrointestinal digestion while IWH further generated WH after transepithelial transport [7,12]; IWH and IW possess antihypertensive, antioxidant, and anti-inflammatory activity while WH is an anti-atherosclerotic peptide [12,13]. Under these circumstances, the activity of the parent peptides is determined by the fragments formed in the gastrointestinal tract.

Another deficiency lies in an incomplete understanding of the mechanisms of action. It was reported that ACE inhibitory peptides possessing significant blood pressure reduction in SHRs did not always inhibit the activity of ACE in vivo, which indicated the possible involvement of other mechanisms of action [6,14,15]. This could be further supported by many studies in which some peptides, possessing much weaker in vitro ACE inhibition than that of captopril, caused comparable blood pressure reduction in SHRs [6,16]. Although the renin-angiotensin-system (RAS) is deemed as the key regulator of blood pressure, many peptides, initially characterized as ACE inhibitory peptides, have not had their in vivo ACE inhibition confirmed [4]; further investigations indicated that their antihypertensive actions involved other mechanisms, such as improvement of vascular inflammation, restoration of nitric oxide-dependent vasorelaxation, and upregulation of angiotensin-converting enzyme 2 (ACE2, an ACE homologue that degrades Ang II), among others [17]. Hence, ACE inhibition is not the only index to screen and evaluate the antihypertensive potential of a hydrolysate or a peptide. For example, we previously identified a potent ACE inhibitory peptide IRW (IC_50_ value of 0.64 μM) that reduced blood pressure in SHRs, but no ACE inhibition was detected in vivo. Later, mitigation of vascular inflammation and restoration of endothelium-dependent vasorelaxation mediated by upregulation of ACE2-Ang (1–7)-MasR axis was found to be the major mechanism [18]. Studies have also reported the importance of other antihypertensive-related activities (other than ACE inhibition) in blood pressure control such as antioxidant and anti-inflammatory activities [19,20,21]. These findings prompted us to use other biomarkers, such as ACE2 upregulation, anti-oxidation and anti-inflammation, in addition to ACE inhibition, to study the antihypertensive activity of a hydrolysate or peptide.

Eggs are excellent protein sources and are an important part of the human diet. Global egg production was approximately 76.7 million metric tons in 2018 [22]. Eggs are a well-known source of many bioactive compounds such as bioactive peptides, lysozyme, and immunoglobulin Y [23,24]. One byproduct associated with egg consumption is eggshells, which are usually disposed of mainly by landfills; however, recent value-added applications have focused on utilizing eggshells as absorbents, antimicrobials, and catalysts [25]. Spent hens are another major byproduct of the egg industry; these are the birds that reach the end of their egg-laying cycle. Every year, more than 400,000,000 spent hens are produced in North America [26,27]. Only a small proportion of spent hens are processed as foods or feeds, due to a low meat yield, poor meat quality, and high cost of processing; they are mostly disposed by burial, composting, and incineration, which negatively affects the environment due to excessive carbon emission, and involves animal welfare issues [28,29]**.** Thus, finding a more sustainable solution for spent hen utilization which also yields additional value to the egg industry is highly relevant. Previous work on spent hens has mainly included extraction and characterization of spent hen proteins [28,30], fabrication of nanomaterials [30,31,32], and preparation of bioactive peptides [29,33,34,35,36,37]. We have previously prepared a spent hen muscle protein hydrolysate (SPH) that exhibited anti-inflammatory activity in young rodents [34]; a few anti-inflammatory peptides have been identified therein [29]. We also developed a line of techniques that are effective in producing low-molecular-weight collagen peptides from spent hens [35,38]. Given that meat proteins are excellent sources of antihypertensive peptides [39,40], we continued to explore the antihypertensive effect of SPHs, in order to further develop spent hen proteins as functional food ingredients. In this study, nine enzymes were used to produce SPHs and their in vitro antihypertensive potential was evaluated and screened by a multiple evaluation approach, including ACE inhibitory, ACE2 upregulating, antioxidant and anti-inflammatory activity, coupled with gastrointestinal digestion and transepithelial transport, before administration to SHRs. ACE2 upregulation and antioxidant activity were assessed in rat vascular smooth muscle cells (A7r5); anti-inflammatory activity was evaluated in human endothelial cells (EA.hy926) [41].

## 2. Materials and Methods

### 2.1. Materials

Spent hen (~70–80 weeks old, ~1.12 kg) carcasses were purchased from a local supermarket (T&T) in Edmonton, Alberta, Canada. Thermoase PC10F (from *Bacillus thermoproteolyticus Var. Rokk*, 90,000 U/g protein), Protease S (10,000 U/g) and Protease M (40,000 U/g) were obtained from Amano Enzyme Inc. (Nagoya, Japan). Alcalase 2.4L (2.4 U/g) was bought from Novozymes (Franklinton, NC); Protex 50FP (500,000 U/g), Protex 26L (2000 U/g), and Protex 6L (580,000 U/g)were purchased from Genencor International Inc. (Rochester, NY); pepsin (from porcine gastric mucosa, 1064 U/mg), trypsin (from porcine pancreas, 1983 U/mg)), methane-sulfonic acid, 2,4,6-trinitrobenzenesulfonic acid (TNBS), acetonitrile, trifluoroacetic acid (TFA), ACE (from rabbit lung), N-hippuryl-His-Leu (HHL), cytochrome C, aprotinin, vitamin B_12_, (glycine)_3_, and glycine, were purchased from Sigma-Aldrich (Oakville, ON, Canada). A7r5 (CRL-1444), EA.hy926 (CRL-2922), and Caco-2 (HTB-37) cell lines were obtained from ATCC (Manassas, VA, USA). Dulbecco’s modified Eagle’s medium (DMEM), 0.25% (*w/v*) trypsin-0.53 mM EDTA, fetal bovine serum (FBS), Hanks balanced salt solution (HBSS with Ca and Mg), nonessential amino acids (NEAA), 4-(2−68 hydroxyethyl)-1-piperazineethanesulfonic acid (HEPES), and penicillin−streptomycin were obtained from Gibco Invitrogen (Burlington, ON, Canada).

### 2.2. Preparation of SPHs

Spent hen muscle proteins were extracted from spent hen carcasses using a pH-shift method [30]. Briefly, spent hen muscle was collected after deboning, skinning, and removal of external fat, and was then homogenized (for 2.5 min) in deionized water (ddH_2_O) using a Wearing heavy duty blender (Wearing Commercial, Torrington, CT). The homogenate was left at pH 11.0 (using NaOH) for 30 min for protein dissolution under continuous stirring (500 rpm) before the first centrifugation at 10,000× *g* (20 min, 4 °C). Then, the supernatant was collected and adjusted to pH 5.0 (using HCl) for another 30-min stirring (500 rpm). After the second centrifugation, the precipitate (protein extract) was collected, washed three times (using ddH_2_O), and freeze-dried. The extraction process was repeated three times.

The protein extract (~93% protein) was dissolved in ddH_2_O (5%, *w/w*). After being heated at 90 °C for 10 min for protein denaturation, the slurry was hydrolyzed by nine enzymes in a jacket beaker, connected with a Titrando (Metrohm, Herisan, Switzerland) and a circulating water bath (Brinkman, Mississauga, ON, Canada) for a constant pH and temperature control, respectively. Protein hydrolysates were prepared by using either one (4% enzyme/substrate, E/S, *w/w* protein) or two enzymes (2% E/S for each, *w/w* protein) for 3 h, with hydrolysis parameters depicted in Tables S1 and S2. With regards to two-enzyme hydrolysis, enzymes with similar working pH were added together; while for those with different working pH, the first enzyme was added for 1.5 h followed by the second one for another 1.5 h (without enzyme inactivation in between) under their respective working conditions. After the hydrolysis, the slurry was heated at 95 °C for 10 min to terminate the reaction and then centrifuged at 10,000 g for 15 min at 4 °C. The supernatant was freeze-dried and kept at −20 °C for further analysis. Each hydrolysate was produced in triplicate.

### 2.3. Analysis of Protein Content, Hydrolysis Yield, and Degree of Hydrolysis (DH) of SPHs

Protein content was estimated by converting the nitrogen content (N factor of 6.25) which was determined by a TruSpec CN carbon/nitrogen determinator (Leco Corp., St. Joseph, MI, USA). Dry matter was analyzed by drying the samples at 110 °C overnight. Ash content was determined by placing samples at 550 °C for 24 h. Hydrolysis yield was calculated based on the following formula:

Protein Yield (%) = (protein content of hydrolysate/protein content of protein extract) × 100

DH was determined using the TNBS method [42]. DH was defined as the % of cleaved peptide bonds of the total peptide bonds of the original protein extract; the number of peptide bonds was determined as that of the amino groups of the samples.

### 2.4. Sodium Dodecyl Sulfate-Polyacrylamide Gel Electrophoresis (SDS-PAGE)

Hydrolysates, dissolved in water (10 mg/mL), were diluted in a ratio of 1:1 using a 2× Laemmli sample buffer with 5% β-mecaptoethanol. After heating at 95 °C for 5 min, samples were loaded (20 µL) to a 16.5% Mini-Protean Tris-Tricine gel in a Mini-PROTEAN Tetra Cell with a PowerPac Basic electrophoresis apparatus (Bio-Rad Laboratories, Inc., Hercules, CA, USA) at a constant voltage of 150 V. Gels were stained with Coomassie brilliant blue R250 and de-stained using a solution of ddH_2_O-methanol-acetic acid (5:4:1, *v/v/v*), and were then scanned in Alpha Innotech gel scanner (Alpha Innotech Corp., San Leandro, CA, USA).

### 2.5. Size Exclusion Chromatography

Molecular weight distribution was analyzed by size exclusion chromatography using a Superdex peptide 10/300 GL column (at room temperature), connected with an AKTA explorer 10XT system (GE Healthcare, Uppsala, Sweden). Samples were dissolved in 30% ACN (in ddH_2_O, *v/v*) containing 0.1% TFA. Samples (100 µL, 1 mg/mL) were injected into the column and eluted at an isocratic gradient at flow rate of 0.5 mL/min. Peaks were detected at 220 nm. Cytochrome C, aprotinin, vitamin B_12_, (glycine)_3_, and glycine were used as molecular weight markers.

### 2.6. Desalting Protocol, Simulated Gastrointestinal Digestion, and ACE Inhibitory Assay of SPHs

Prior to treatment with cells, SPHs were desalted according to the protocol described in Fan et al. [43]. Briefly, samples were dissolved in ddH_2_O and loaded onto the Sep-Pak 35cc tC18 cartridge (WAT043350, Waters, Milford, MA, USA). The cartridge was first washed with two column volumes (CV) of ddH_2_O for salt removal. Then 50% ACN (2 CV) and 100% ACN (1 CV) were added sequentially to wash the cartridge; the ACN eluent was collected, vacuum evaporated, and freeze-dried.

Simulated gastrointestinal digestion was performed as described in Fan et al. [43]. Briefly, SPHs (5% protein in ddH_2_O, *w/v*) were digested by pepsin (1% E/S, *w/w* protein) for 1.5 h at 37 °C, pH 2.0 (adjusted with 3 M HCl). Then, the digest was adjusted to pH 7.5 with 1 M NaOH, half of the digest collected as pepsin digest and another half further digested by pancreatin (1% E/S, *w/w* protein) for 1.5 h (at 37 °C). Reaction was terminated by heating the digests to 95 °C (maintained for 10 min).

Determination of ACE inhibition of SPHs followed the procedures described in Fan et al. [43]. The IC_50_ value was defined as the sample concentration inhibiting 50% of ACE activity.

### 2.7. Cell Culture of A7r5, EA.hy926, and Caco-2 Cells

Cell culture protocol of A7r5 (passages 4–11), EA.hy926 (passages 3–10), and Caco-2 (passages 22–28) cell lines referred to our previous studies [7,9,44,45]. Cells were grown in DMEM supplemented with 10% FBS, 25 mM HEPES, and 1% antibiotics (penicillin-streptomycin) at 37 °C in a 100% humidified atmosphere with 5% CO2; nonessential amino acids (1%) were supplemented for EA.hy926 and Caco-2 cell lines. The growth media were changed every three days for A7r5 and EA.hy926 cells and every 2 days for Caco-2 cells.

### 2.8. Western Blotting

The confluent A7r5 cells were placed in a quiescing medium (the same as that of the growth medium but with 1% FBS). A7r5 cells were treated with 2.5 mg/mL of SPHs for 24 h for ACE2 detection. EA.hy926 cells were treated with 2.5 mg/mL of SPHs for 18 h prior to the addition of 10 ng/mL of tumor necrosis factor-alpha (TNFα) for a 6 h co-treatment for detection of intracellular adhesion molecule-1 (ICAM-1) and vascular cell adhesion molecule-1 (VCAM-1). The dose of hydrolysate was selected based on our previous studies [9]. Cells were lysed in boiling Laemmle’s buffer containing 50 mM Dithiothreitol (DTT) and 0.2% Triton-X-100.

Cell lysates were loaded onto a 9% separating gel and transferred to a nitrocellulose membrane (diameter 0.45 µm, 1620115, Bio-Rad, Montreal, QC, Canada) for incubation with antibodies. Bands of ACE2 (ab87436, Abcam, Toronto, ON, Canada), ICAM-1 (sc-8439, Santa Cruz, Dallas, TX, USA) and VCAM-1 (sc-8304, Santa Cruz, Dallas, TX, USA) were normalized to α-tubulin (ab15246, Abcam). Donkey-anti-rabbit 800 CW or donkey-anti-mouse IRDye 680 RD secondary antibodies (Licor Biosciences, Lincoln, NE, USA) were used to visualize the fluorescent bands in a Licor Odyssey BioImager, which were quantified using Image Studio Lite 5.2 (Licor Biosciences).

### 2.9. Superoxide Detection

Superoxide generation in A7r5 cells was detected by dihydroethidium (DHE) staining, as described by Wang et al. [46] with slight modifications. The cells were treated with 2.5 mg/mL of SPHs for 1 h prior to the addition of 1 μM of Ang II for 0.5 h. Then, DHE (20 μM) was added and incubated with cells for 30 min (protected from light). Later, the cells were washed three times with a non-phenol-red DMEM (21063029, Thermo Fisher Scientific, Burlington, ON, Canada). The fluorescence signal was detected by an Olympus IX81 fluorescent microscope (Olympus, Tokyo, Japan). Each data point was taken from three random fields. The total fluorescence intensity in each field was quantified using ImageJ software (https://imagej.net/Welcome) and the mean fluorescence intensity per cell (MFI/cell) was determined. Results were expressed as fold change of the Ang II-treated group; the untreated group was without any hydrolysates or Ang II treatment.

### 2.10. Caco-2 Transport of the Gastrointestinal-Digested SPH

Preparation of trans-well plates and transport experiments followed the procedures described in Fan et al. [7] and Liang et al. [9]. After cells were seeded for one week, transepithelial electrical resistance (TEER) was monitored every two days using an ohmmeter (World Precision Instruments, Sarasota, FL, USA), and, on day 21, only wells with TEER values >400 Ω/cm^2^ were used. The gastrointestinal digest of SPH-T (20 mg/mL) was dissolved in HBSS, pre-warmed, and added to the apical chambers (0.5 mL); the permeates in the basolateral chambers (1.5 mL) were collected for up to 4 h. Only samples in wells with TEER values >400 Ω/cm^2^ after transport study were used for later analysis. Peptide concentrations were determined by the Modified Lowry Protein Assay Kit (Thermo Fisher Scientific, Burlington, ON, Canada). The permeability of transport was expressed as the % (*w/w*) of peptides transported. Chromatograms of the samples before and after transport were analyzed using an Acquity BEH C18 column (1.7 μm, 2.1 × 100 mm) on a reverse-phase ultra-performance liquid chromatography (RP-UPLC) system. Samples (10 μL) were eluted using a gradient of chromatographic grade H_2_O and ACN (both containing 0.05% TFA) at 0.3 mL/min as follows: 1% B (0–3 min) and 1–23% B (3–28 min); absorbance was monitored at 220 nm.

### 2.11. Identification of Peptides of Caco-2 Permeates

The major peak of Caco-2 permeate of gastrointestinal-digested SPH-T was analyzed by liquid chromatography tandem mass spectrometry (LC-MS/MS). The permeate after Caco-2 transport (5 μL, desalted) was injected and linearly eluted (0.3 mL/min): 1–60–95% B (0–2–40–55 min) through a nanoAcquity UPLC system, connected with an Atlantis dC_18_ UPLC column (75 μm × 150 mm, 3 μm, Waters) and a micro-mass quadrupole time-of-flight (Q-TOF) premier mass spectrometer. Solvents were chromatographic grade H_2_O (solvent A) and ACN (solvent B) both containing 1% formic acid. Ionization was performed using electrospray ionization technique in a positive ion mode (capillary voltage, 3.4 kV; source temperature, 100 °C). Peptide mass was detected using a Q-TOF analyzer; acquisition ranges were m/z 200–1200 (MS mode) and 50–1990 (MS/MS mode), respectively. Data were interpreted by MassLynx software version 4.1 (Waters) by de novo sequencing.

### 2.12. Cytotoxicity

Cytotoxicity of SPHs against A7r5, EA.hy926, and Caco-2 cells followed the alamarBlue fluorescence assay provided by Thermo Fisher Scientific (Burlington, ON, Canada). Cells were seeded on a 96-well plate at 1.0 × 10^4^ cells/well. After reaching 80% of confluency, cells were treated with SPHs (dissolved in culture medium) for 24 h. Then, culture media were replaced with 200 μL of 10% alamarBlue solution (dissolved in culture medium) for 4 h of incubation (protected from direct light), after which 150 μL was transferred into an opaque 96 well plate for detection of fluorescence signal; emission and excitation wavelengths were at 590 nm and 560 nm, respectively. The control was without any treatment. The concentration used was 2.5 mg/mL for A7r5 and EA.hy926 cells and 20 mg/mL for Caco-2 cells [9].

### 2.13. Ethics Statement, Animal Model and Telemetry Recording

The animal protocol (#AUP 00001571) was approved by the Animal Welfare Committee at the University of Alberta following the guidelines issued by the Canadian Council on Animal Care and adhered to the Guide for the Care and Use of Laboratory Animals published by the United States National Institutes of Health. Twelve- to fourteen-week-old SHRs (290 ± 10 g) were obtained from the Charles River (Senneville, QC, Canada). Upon arrival, rats were acclimatized in the university animal core facility, fed with standard rat chow and water ad libitum and exposed to a 12:12 h of light: dark cycle under controlled humidity and temperature. After one week, they were surgically implanted with telemetry transmitters (HD-S10, Data Sciences International, St. Paul, MN, USA) as previously described by Majumder et al. [47] and Fan et al. [7] with slight modifications. To reduce stress in animals, the transmitter was placed in the left groin area instead of being placed on the left hip area, with the catheter inserted into the common femoral artery and advanced into the abdominal aorta; the catheter was secured to the vessel using a vicryl 5-0 suture. After surgery, the rats were caged individually and allowed a 7-day postoperative recovery.

Rats were randomly assigned into four groups (*n* = 3): untreated and three hydrolysate groups (SPHs digested by thermoase, pepsin, and Protex 26L). Hydrolysates were dissolved in 10 mL of Ensure (Abbott Nutrition, QC, Canada) and administrated orally to rats at a daily dose of 1000 mg/kg body weight (BW) once per day from day 1; untreated group was given Ensure only. The dose was selected based on our previously-reported studies [19,43]. Mean arterial pressure (MAP) was recorded over a continuous 24 h (10 s of every 1 min) every two days until day 20 (blood pressure on day 0 was recorded as the baseline). At the end of the experiment, animals were sacrificed by cardiac blood collection under anesthesia.

### 2.14. Statistical Analysis

Data of hydrolysis yield, protein content, DH, and ACE inhibition of SPHs were performed in triplicate and were analyzed using one-way analysis of variance (ANOVA) by IBM SPSS Statistics Version 23 (Chicago, IL, USA) followed by Duncan’s multiple range tests. Data from the cell study were expressed as means ± standard errors (SEMs) of four independent experiments (except for cytotoxicity, replicated six times) and were analyzed by one-way ANOVA followed by Dunnett’s multiple test using GraphPad Prism version 6 (San Diego, CA, USA). Blood pressure (means ± SEMs) was analyzed by two-way ANOVA followed by Tukey’s test using GraphPad. Significant level was set as 5%.

## 3. Results

### 3.1. Hydrolysis Yield, Protein Content, DH, and ACE Inhibition of SPHs

Table 1 shows hydrolysis yield, protein content, DH, and in vitro ACE inhibition of SPHs prepared by nine individual enzymes. SPHs digested by pepsin (SPH-P) and Protex 26L (SPH-26L) showed the highest ACE inhibition, with their respective IC_50_ values of 23 ± 0.9 and 24 ± 1.5 μg/mL, followed by SPH digested by thermoase (SPH-T, 30 ± 1.4 μg/mL). Thermoase, alcalase, and Protex 26L produced SPHs with the highest hydrolysis yield of 86.5 ± 1.4%, 79.8 ± 4.0%, and 76.4 ± 1.5%, respectively. Trypsin, protease M and thermoase produced SPHs with the highest protein content of 83.4 ± 2.2%, 81.0 ± 0.7%, and 79.2 ± 1.2%, respectively. SPH produced by Protease M had the highest DH (32.7 ± 0.2%), followed by alcalase (22.1 ± 2.3%), thermoase (21.9 ± 0.1%), and Protex 26L (20.6 ± 0.6%).

To study whether or not ACE inhibition could be further improved by a combination of enzymes, enzymes with similar hydrolysis temperatures and pHs were then combined to prepared two-enzyme digested SPHs (Table S2); Trypsin and Protease M were excluded due to their low potential in producing ACE inhibitory peptides (Table 1). As shown in Table S3, ACE inhibitory activity was not further improved by enzyme combinations. Therefore, pepsin, Protex 26L, and thermoase showed the best capability in producing ACE inhibitory peptides.

### 3.2. Molecular Weight Distribution of SPHs

**Figure 1:**
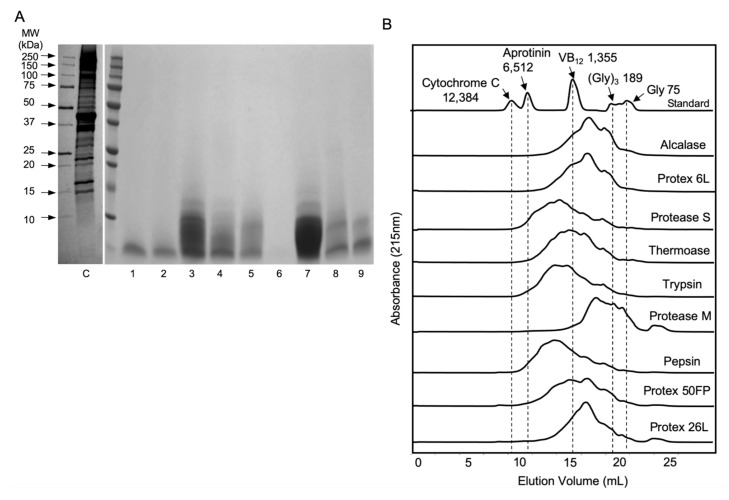
SDS-PAGE (**A**) and size exclusion chromatogram (**B**) of one-enzyme digested SPHs. C–Control (non-hydrolyzed sample); Lane 1–9: 1-Alcalase, 2-Protex 6L, 3-Protease S, 4-Thermoase, 5-Trypsin, 6-Protease M, 7-Pepsin, 8-Protex 50FP, 9-Protex 26L. VB_12_, vitamin B12; Gly, glycine. Values (75–12,384) in Figure B indicate the molecular weight (Da) of Gly, (Gly)_3_, VB_12_, aprotinin, and cytochrome C.

SDS-PAGE analysis was applied to study the effect of hydrolysis on spent hen meat proteins. As shown in Figure 1A, almost all enzymes hydrolyzed spent hen muscle proteins into peptides below 10 kDa. Protease M showed the highest efficiency in the hydrolysis, followed by that of Protex 6L, alcalase, thermoase, trypsin, Protex 50FP, and Protex 26L, while protease S and pepsin were the least efficient. Then size exclusion chromatography was applied to further determine the molecular weight distribution of these hydrolysates (Figure 1B). Pepsin and protease S had the lowest efficiency while protease M had the highest efficiency in preparing low-molecular-weight peptides.

### 3.3. ACE2 Upregulating, Antioxidant, and Anti-Inflammatory Activity of SPH-P, SPH-26L, and SPH-T

Since SPH-P, SPH-26L, and SPH-T possessed the highest ACE inhibitory activity while also contained a high proportion of low-molecular-weight peptides, their ACE2 upregulating, antioxidant, and anti-inflammatory activities in A7r5 cells or EA.hy 926 cells were further studied. All three SPHs showed no cytotoxicity against both cells (Figure S1A,B). As shown in Table 2, only SPH-T upregulated ACE2 expression (*p* < 0.05) while all three SPHs diminished Ang II-induced oxidative stress in A7r5 cells. All three SPHs also reduced expressions of VCAM-1 (*p* < 0.05) but not ICAM-1 in EA.hy 926 cells. These SPHs were further tested against gastrointestinal digestion.

### 3.4. Effect of Gastrointestinal Digestion on ACE Inhibitory, ACE2 Upregulating, Antioxidant, and Anti-Inflammatory Activity of SPH-P, SPH-26L, and SPH-T

**Figure 2:**
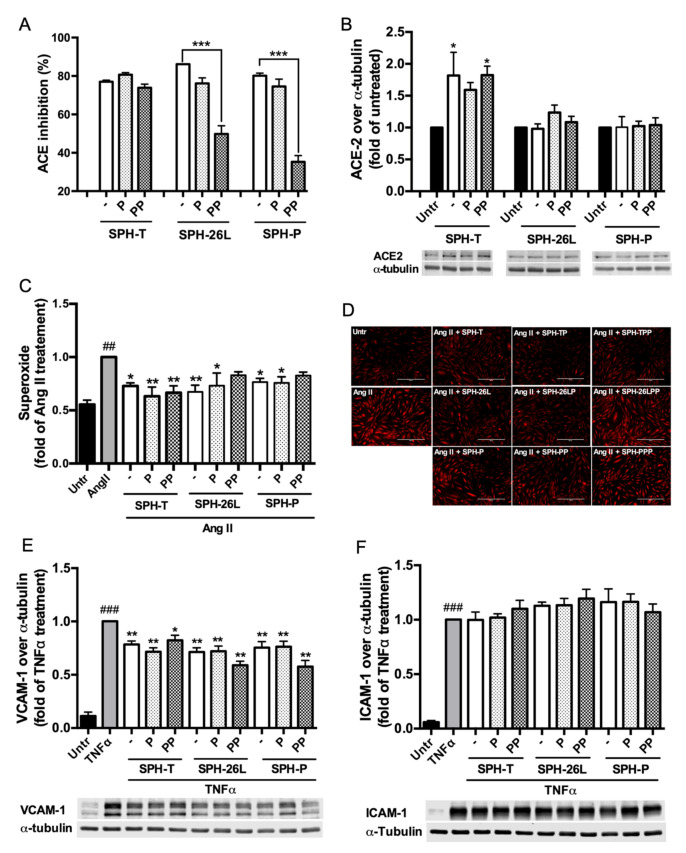
Effect of gastrointestinal digestion on ACE inhibitory, ACE2 upregulating, antioxidant, and anti-inflammatory activities of spent hen hydrolysates. SPH-T, SPH-P, and SPH-26L refer to spent hen hydrolysates prepared respectively by thermoase, pepsin, and Protex 26L. -, P, and PP indicates non-, pepsin-, and pepsin plus pancreatin digestion, respectively (*n* = 4). (**A**) In vitro ACE inhibition (***, *p* < 0.001). (**B**) ACE2 expression in A7r5 cells (*, *p* < 0.05, compared to untreated group). (**C**,**D**) Oxidative stress in A7r5 cells (##, *p* < 0.01, compared to untreated group; *, *p* < 0.05, **, *p* < 0.01, compared to Ang II-treated group). (**E**,**F**) ICAM-1 and VCAM-1 expressions in EA.hy926 cells (###, *p* < 0.001 compared to untreated group; *, *p* < 0.05, **, *p* < 0.01, compared to TNFα-treated group). ACE inhibition was determined at 0.125 mg/mL of SPHs while cells were treated with 2.5 mg/mL of SPHs.

ACE inhibitory activity of SPH-T was not affected while those of SPH-26L and SPH-P were reduced markedly (*p* < 0.001) after simulated gastrointestinal digestion (Figure 2A). ACE2 expression induced by all three SPHs was not affected after the digestion (Figure 2B). Antioxidant activity of SPH-T was maintained while those of SPH-26L and SPH-P were reduced after the digestion (Figure 2C,D). All three SPHs mitigated TNFα-induced upregulation of VCAM-1 in EA.hy926 cells but did not affect ICAM-1 expression throughout the digestion (Figure 2E,F). SPH-T after peptic and pancreatic digestion (SPH-TPP) was selected to further determine its stability and permeability across Caco-2 monolayers.

### 3.5. Effect of Caco-2 Transport on ACE Inhibitory, ACE2 Upregulating, Antioxidant, and Anti-Inflammatory Activities of SPH-TPP (pepsin-pancreatin digested spent hen hydrolysate prepared by thermoase)

**Figure 3:**
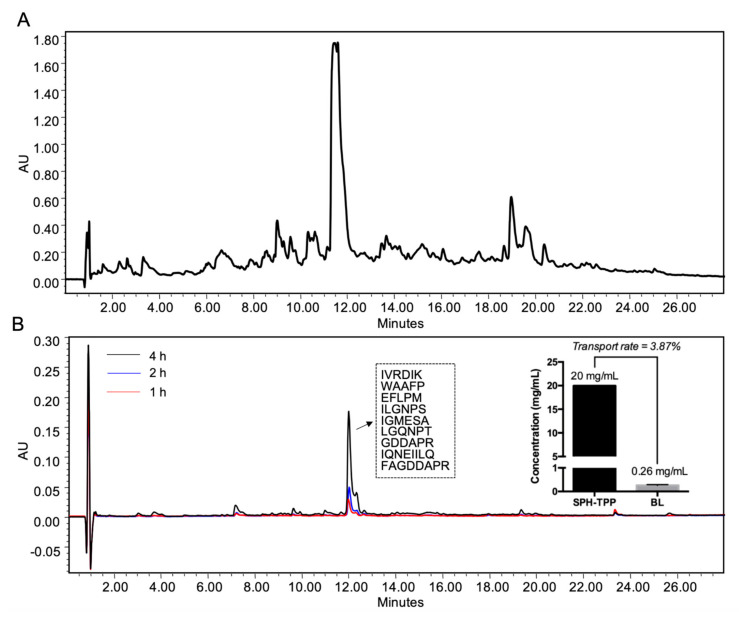
Chromatograms of SPH-TPP (**A**) and its permeate (**B**) after 4 h of transport across Caco-2 cell monolayers (*n* = 4). Transport permeability (at 4 h) was based on the % of peptides transported to the basolateral (BL) side. Peptide sequences of the major peak after transport are inserted. SPH-TPP, pepsin-pancreatin digested spent hen hydrolysate prepared by thermoase.

**Figure 4:**
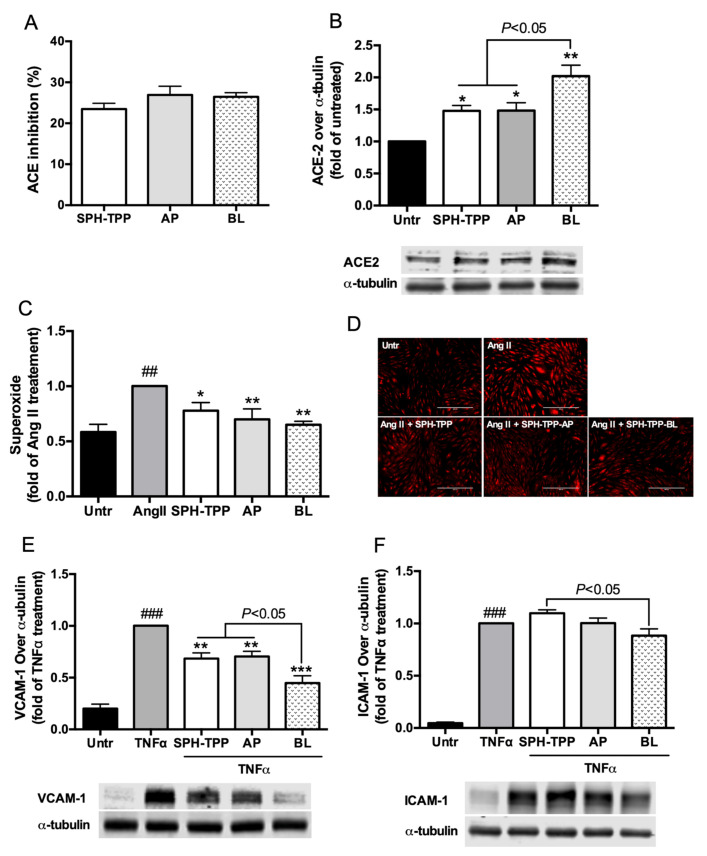
Effects of transport across Caco-2 cell monolayers on ACE inhibitory, ACE2 upregulating, antioxidant, and anti-inflammatory activities of SPH-TPP (*n* = 4). SPH-TPP, pepsin-pancreatin digested spent hen hydrolysate prepared by thermoase; AP, apical side; BL, basolateral side. (**A**) In vitro ACE inhibition. (**B**) ACE2 expression in A7r5 cells (*, *p* < 0.05, **, *p* < 0.01, compared to untreated group). (**C**,**D**) Oxidative stress in A7r5 cells (##, *p* < 0.01 compared to untreated group; *, *p* < 0.05, **, *p* < 0.01 compared to Ang II-treated group). (**E**,**F**) ICAM-1 and VCAM-1 expression in EA.hy926 cells (###, *p* < 0.001 compared to untreated group; **, *p* < 0.01, ***, *p* < 0.001 compared to TNFα-treated group). ACE inhibition was determined at 16 μg/mL of SPHs while cells were treated with 2.5 mg/mL of SPHs.

SPH-TPP did not show any cytotoxicity against Caco-2 cells (Figure S1C). Figure 3 presents the chromatographic profiles of SPH-TPP before and after the transport. The permeability at 4 h was 3.87 ± 0.58% based on the peptide transported (the permeability within 4 h is shown in Figure S2). The transport process did not affect ACE inhibition of SPH-TPP (Figure 4A). ACE2 upregulating and antioxidant activities in A7r5 cells by SPH-TPP were further improved after transport (Figure 4B,D). Its ability in attenuating VCAM-1 expression in TNFα-induced EA.hy926 cells was improved (*p* < 0.05), while the inhibition of ICAM-1 expression was unexpectedly enhanced after the transport, despite not being significantly compared to the TNFα-treated group (Figure 4E,F). Nine major peptide sequences (IVRDIK, WAAFP, EFLPM, ILGNPS, IGMESA, LGQNPT, GDDAPR, IQNEIILQ, and FAGDDAPR) were identified from the major peak of the basolateral eluent of SPH-T (its mass spectrum is shown in Figure S3).

### 3.6. Antihypertensive Effect of SPHs in SHRs

**Figure 5:**
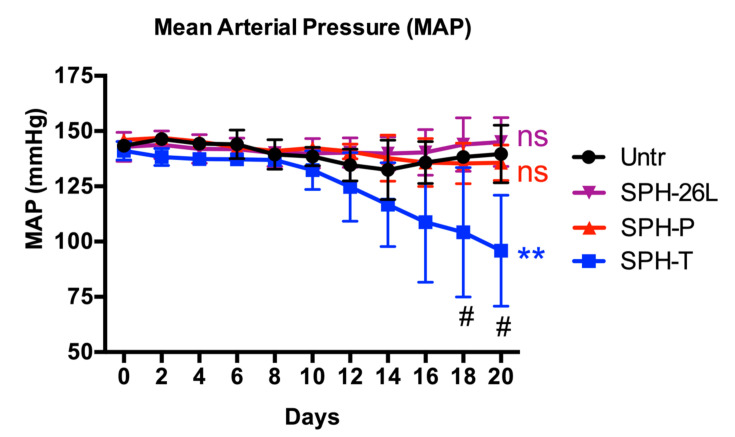
Effects of SPH-T, SPH-26L, and SPH-P on MAP (mean arterial pressure, mmHg) in SHRs (*n* = 3). SPH-T, SPH-P, and SPH-26L refer to spent hen hydrolysates prepared respectively by thermoase, pepsin, and Protex 26L. SPHs were orally administrated at 1000 mg/kg BW per day for a continuous 20 days. Each data point was represented as the MAP value recorded over a 24-h period and was expressed as means ± standard errors of means. #, *p* < 0.05, compared to untreated group (Untr, without any SPH) at the time point. **, *p* < 0.01, ‘ns’, not significant, compared to untreated group over the entire treatment period.

SPH-T was orally administrated to SHRs (at 1000 mg/kg BW per day) to explore its in vivo activity; SPH-P and SPH-26L were also administrated to SHRs due to their high in vitro ACE inhibitory activities (Figure 5). Only SPH-T reduced blood pressure after a period of 20 days, with MAP lowered from 141.1 ± 4.2 mmHg to 95.9 ± 25.1 mmHg (*p* < 0.01). SPH-P and SPH-26L did not affect blood pressure throughout the treatment period.

## 4. Discussion

Our study reported the preparation of bioactive peptides from spent hen muscle proteins. Hydrolysis yield, protein content and DH of SPHs varied significantly among different enzymes (Table 1 and Table S3). Compared to one-enzyme hydrolysis, use of two enzymes further increased protein content of SPHs, since a combination of enzymes could contribute to broader cleavage sites to some extent [43]; however, this did not further enhance ACE inhibitory activity of SPHs. There was no obvious correlation between DH and ACE inhibition of SPHs. Among SPHs obtained, SPH-P, SPH-26L, and SPH-T possessed the highest ACE inhibition, with IC_50_ values of 23–30 μg/mL (Table 1). Their ACE inhibitory activities were higher than many hydrolysates of other sources such as casein, albumin, ovalbumin, ovotransferrin, and egg white [11,24,43,48]. The ACE inhibitory activity of peptides is dictated by their amino acid sequences, which are dependent largely on proteases, protein sequences, and the conditions applied to release the peptides [4]. Indeed, ACE inhibitory activities of SPHs varied significantly among different proteases, which was due to their different cleavage specificities (Table 1). Meat proteins have been reported to be particularly rich in antihypertensive peptides [39,40]. Our previous study demonstrated that chicken muscle proteins are excellent sources of ACE inhibitory peptides, superior to those of many others such as milk, egg, soybean, and fish [49]; this might, to some extent, explain the potent ACE inhibitory activity of SPHs. Our results further supported a great potency of chicken muscle proteins as a raw material for production of antihypertensive peptides. Compared with protein hydrolysates from other meat byproducts such as skin, bone, viscera, blood and sarcoplasmic proteins, SPHs appeared to have a more potent ACE inhibitory activity (IC_50_ values of 23–189 μg/mL) [50,51,52,53,54]. The extracted proteins in this study contained mainly myofibrillar proteins as reported previously [29,30]. The major protein bands include myosin heavy chain (~220 kDa), actin (42 kDa), and myosin light chain (15–25 kDa) (Figure 1A) [37,55,56]. These proteins were cleaved into peptides below 10 kDa after the hydrolysis and more than half of peptides were below 3 kDa (Figure 1A,B). Therefore, based on the results of hydrolysis profiles and ACE inhibition, three hydrolysates, SPH-P, SPH-26L, and SPH-T, were selected for study of ACE2 upregulating, antioxidant, anti-inflammatory activities in the two vascular cells.

Vascular smooth muscle and endothelial cells are two important components of the blood vessel wall that collaboratively maintain vascular homeostasis and regulate blood pressure [57,58]. Hypertension is associated with endothelial dysfunction and vascular remodelling that, at the cellular level, feature in many pathological responses such as oxidative stress, inflammation, and migration [57,59,60]. A7r5 and EA.hy926 cells are two well-established models of evaluating cellular antioxidant and anti-inflammatory activity of bioactive peptides, respectively [41]; ACE2 upregulation in A7r5 cells has recently been used to evaluate the antihypertensive potential of bioactive peptides [17,18,46]. For example, IRW, derived from egg white, and LRW, derived from pea, both upregulated ACE2 in A7r5 cells by approximately two times [46,61]. Blood pressure reduction is accompanied with ACE2 upregulation in various tissues in SHRs such as heart, kidney, aorta, and mesenteric arteries [16,21,61,62,63]. SPH-T enhanced ACE2 expression in A7r5 cells, indicating its potential antihypertensive ability in vivo. SPH-T, SPH-26L, and SPH-P all diminished oxidative stress in Ang II-induced A7r5 cells (Table 2), consistent with the fact that muscle proteins are rich in antioxidant peptides [64]. Many other meat byproduct-derived protein hydrolysates have shown in vitro antioxidant activity such as those prepared using bone, blood, and skin; however, these hydrolysates have rarely been studied on cellular antioxidant activity which is thought to be more biologically relevant [64]. All three SPHs inhibited expression of VCAM-1 (*p* < 0.05) to a similar extent but did not affect that of ICAM-1 in TNFα-induced EA.hy926 cells. Hydrolysates or peptides with anti-inflammatory effects have been prepared from various food commodities such as meat, zein, beans and egg white, some of which have further been validated their physiological efficacy in animals [9,29,34,47,65,66]. Next, we studied the effects of simulated gastrointestinal and transport digestions on the activities of SPH-T, SPH-26L, and SPH-P.

Gastrointestinal digestion did not affect ACE inhibitory, ACE2 upregulating, antioxidant, and anti-inflammatory activities of SPH-T, but reduced those of SPH-26L and SPH-P mainly in ACE inhibitory and antioxidant activities (Figure 2). These results suggested a different susceptibility of three hydrolysates during gastrointestinal digestion. Since many potent chicken meat-derived ACE inhibitory peptides contain lysine or arginine residues, they are good substrates of trypsin [49]. This explained a decline in ACE inhibitory activity of these hydrolysates after pancreatic digestion. Previous research indicated that peptides exert cellular antioxidant activity through either acting as direct scavengers or activating cellular signaling such as the nuclear factor erythroid 2−related factor 2 pathway, demonstrating that antioxidant activity of peptides depends both on their amino acid compositions and sequences [20,46,65,67,68]. A higher resistance to the gastrointestinal digestion of SPH-T than those of SPH-26L and SPH-P might be responsible for its more retained antioxidant activity after the gastrointestinal digestion (Figure 2). We also observed that ACE inhibitory activity of SPH-T was retained to a greater extent than those of SPH-26L and SPH-P. Indeed, many ACE inhibitory peptides have also been reported to be antioxidant peptides [12,46,65,69], indicating that these two types of peptides might share more common sequences in SPH-T. Nevertheless, SPH-T showed good resistance to gastrointestinal digestion and was further subjected to permeability study in Caco-2 cell monolayers. The permeability of SPH-TPP was 3.87 ± 0.58% after 4 h of incubation with Caco-2 monolayers, higher than that of a zein hydrolysate (1.2 ± 0.2%), which was prepared by a sequential hydrolysis by thermolysin, pepsin, and pancreatin [9]. Bioactive peptides have a permeability of generally less than 1% [70]. A relatively higher permeability in our study was possibly due to a high proportion of low-molecular-weight peptides in the SPH-TPP, resulting from a triple digestion by thermoase, pepsin, and pancreatin. This assumption was supported by the existence of more than half of peptides in SPH-T being below 1355 Da (Figure 1B). The transport process did not affect ACE inhibition, but enhanced ACE2 upregulating, antioxidant, and anti-inflammatory activities, indicating a possible further degradation during transport. For example, the basolateral digest significantly (*p* < 0.05) inhibited TNFα-induced VCAM-1 and ICAM-1 expressions compared to that of SPH-TPP, similar to findings reported by Liang et al. [9]. There are numerous peptidases in Caco-2 cells that may contribute to the formation of new peptides during the transport study. In this study, nine peptides (5–8 amino acid residues) were identified from the major Caco-2 permeate of SPH-TPP; future studies are warranted to characterize these peptides in ACE inhibiting, ACE2 upregulating, antioxidant, and anti-inflammatory activities. Nevertheless, the high permeability of SPH-TPP with enhanced bioactivity indicated a likely high bioavailability and in vivo efficacy. The animal study confirmed that SPH-T, but not SPH-26L and SPH-P, was able to significantly reduce the blood pressure of SHRs (Figure 5), despite SPH-26L and SPH-P having higher in vitro ACE inhibitory activity than that of SPH-T. This implied that in vitro ACE inhibition was not always a reliable indicator for screening a hydrolysate for in vivo study; a combination of in vitro activity measurement with gastrointestinal stability and transepithelial permeability is likely to provide a more reliable index for its in vivo activity.

## 5. Conclusions

In this study, a spent hen protein hydrolysate with antihypertensive activity was prepared. Spent hen proteins were hydrolyzed by nine enzymes individually or in combinations; although SPH-26L and SPH-P showed comparable or better ACE inhibitory activity than that of SPH-T, only SPH-T significantly reduced blood pressure in SHR after oral administration. Our study suggested that stability during gastrointestinal digestion and permeability in Caco-2 cells of a hydrolysate are important for in vivo activity, rather than in vitro activity. Our results advocated the use of this multiple evaluation approach in evaluating the antihypertensive potential of a hydrolysate or peptide. Future work is needed to explore the antihypertensive mechanisms of SPH-T in SHRs to better understand the contribution of the above-mentioned activities; furthermore, the identified peptides from the Caco-2 permeate of SPH-TPP need to be characterized. This study supported that spent hen is a valuable raw material for preparing functional food ingredients with antihypertensive activity.

## Figures and Tables

**Table 1:** Effect of single enzyme on hydrolysis yield, protein content, DH, and ACE inhibitory activity of spent hen protein muscle hydrolysates.

Enzymes	Hydrolysis Yield (%)	Protein Content (%)	DH (%)	ACE Inhibition (%) *
				(IC_50_, μg/mL)
Alcalase	79.8 ± 4.0 ^ab^	70.0 ± 1.0 ^c^	22.1 ± 2.3 ^b^	52.5 ± 1.1 (57 ± 2.4) ^d^
Protex 6L	65.0 ± 5.6 ^cd^	71.8 ± 2.4 ^bc^	17.3 ± 0.9 ^c^	42.8 ± 2.2 (79 ± 1.7) ^e^
Protease S	47.8 ± 4.2 ^e^	73.6 ± 2.1 ^bc^	12.2 ± 1.3 ^d^	59.8 ± 1.0 (39 ± 0.5) ^c^
Thermoase	86.5 ± 1.4 ^a^	79.2 ± 1.2 ^a^	21.9 ± 0.1 ^b^	64.8 ± 0.9 (30 ± 1.4) ^b^
Trypsin	44.4 ± 0.1 ^e^	83.4 ± 2.2 ^a^	11.2 ± 0.0 ^d^	18.6 ± 2.7 (189 ± 4.7) ^g^
Protease M	64.5 ± 3.9 ^cd^	81.0 ± 0.7 ^a^	32.7 ± 0.2 ^a^	39.1 ± 0.2 (118 ± 5.0) ^f^
Pepsin	68.6 ± 0.8 ^c^	73.6 ± 1.5 ^bc^	12.2 ± 0.7 ^d^	70.8 ± 0.2 (23 ± 0.9) ^a^
Protex 50FP	58.4 ± 2.1 ^d^	74.7 ± 0.6 ^b^	16.3 ± 2.2 ^c^	52.7 ± 0.2 (50 ± 1.6) ^d^
Protex 26L	76.4 ± 1.5 ^b^	73.8 ± 2.2 ^bc^	20.6 ± 0.6 ^b^	69.5 ± 0.7 (24 ± 1.5) ^a^

* ACE inhibition (%) was determined at 0.05 mg/mL of the hydrolysate (their respective IC_50_ values were shown in the parenthesis). Values (means ± standards deviations) that do not share a common superscript lowercase letter within a column differ significantly (*p* < 0.05) (*n* = 3).

**Table 2:** ACE2 upregulating, antioxidant, and anti-inflammatory activities of spent hen hydrolysates.

Samples	A7r5 Cells		EA.hy926 Cells	
	ACE2	Oxidative Stress	ICAM-1	VCAM-1
	Untreated	Ang II (+)	TNFα (+)	TNFα (+)
SPH-T	1.82 ± 0.36 *	0.73 ± 0.06 *	1.02 ± 0.08	0.80 ± 0.03 *
SPH-P	1.01 ± 0.17	0.77 ± 0.07 *	1.05 ± 0.13	0.73 ± 0.04 *
SPH-26L	0.98 ± 0.08	0.67 ± 0.13 *	1.10 ± 0.04	0.74 ± 0.07 *

ACE2 expression induced by SPHs in A7r5 cells was normalized to that of untreated group (*n* = 4); oxidative stress in A7r5 cells was normalized to that of Ang II-treated group (1 μM) (*n* = 4). ICAM-1/VCAM-1 levels in EA.hy926 cells were normalized to that of TNFα-treated group (10 ng/mL) (*n* = 4). *, *p* < 0.05, indicating a difference compared to the untreated (ACE2), Ang II-treated (oxidative stress), or TNFα-treated (ICAM-1/VCAM-1) group, respectively. SPH-T, SPH-P, and SPH-26L refer to spent hen hydrolysates prepared respectively by thermoase, pepsin, and Protex 26L.

## Supplementary Materials

The following are available online at https://www.mdpi.com/2304-8158/9/10/1384/s1. Table S1: Working parameters of spent hen muscle protein hydrolysis by an individual enzyme. Table S2: Working parameters of spent hen muscle protein hydrolysis by two enzymes. Table S3: Hydrolysis yield, protein content, and DH of two-enzyme digested SPHs. Figure S1: Cytotoxicity of SPHs in A7r5, EA.hy926, and Caco-2 cells. Figure S2: Caco-2 transport of SPH-TPP up to 4 h. Figure S3: mass spectrum of Caco-2 permeate of SPH-TPP.
