# Supplementary material for: Spent Hen Protein Hydrolysate with Good Gastrointestinal Stability and Permeability in Caco-2 Cells Shows Antihypertensive Activity in SHR
Source: Foods. 2020 Oct 1;9(10):1384. doi: 10.3390/foods9101384 (PMC7601532; doi:10.3390/foods9101384)
Supplement: Supplementary file 1 [file foods-09-01384-s001.pdf]

Supplementary materials:

Table S1 Working parameters of spent hen muscle protein hydrolysis by an individual enzyme *

Enzyme	Parameters		
	pH	Temperature (°C)	E/S (%, *w/w*)
Alcalase	8	50	4%
Protex 6L	8	37	4%
Protease S	8	37	4%
Thermoase	8	60	4%
Trypsin	8	60	4%
Protease M	8	60	4%
Pepsin	2	60	4%
Protex 50FP	3	60	4%
Protex 26L	3	60	4%

*3 h hydrolysis. E/S (%, w/w) was based on protein mass.

Table S2 Working parameters of spent hen muscle protein hydrolysis by two enzymes *

Enzyme 1	Parameters			Enzyme 2	Parameters		
	pH	Temperature (°C)	E/S (%, *w/w*)		pH	Temperature (°C)	E/S (%, *w/w*)
Alcalase	8	60	2%	Protex 6L	8	60	2%
Alcalase	8	60	2%	Protease S	8	60	2%
Alcalase	8	60	2%	Thermoase	8	60	2%
Protex 6L	8	60	2%	Protease S	8	60	2%
Protex 6L	8	60	2%	Thermoase	8	60	2%
Protease S	8	60	2%	Thermoase	8	60	2%
Protex 50FP	3	50	2%	Protex 26L	3	50	2%
Protex 50FP	3	50	2%	Pepsin	2	37	2%
Protex 26L	3	50	2%	Pepsin	2	37	2%

*3 h hydrolysis: two enzymes together for 3 h, except for 50FP+pepsin and 26L+pepsin, which were hydrolyzed by the first enzyme for 1.5 h, and then the second enzyme for another 1.5 h without inactivating the first enzyme. E/S (%, w/w) was based on protein mass.

Table S3 Hydrolysis yield, protein content, and DH of two-enzyme digested SPHs

Sample	Hydrolysis yield (%)	Protein content (%)	DH (%)	ACE inhibition (%)*
Alcalase + Protex 6L	77.8±1.0^{a}	87.6±0.1^{cd}	22.0±0.2^{a}	38.0±1.3^{a}
Alcalase + Protease S	76.9±1.6^{ab}	87.4±0.1^{de}	21.4±0.6^{ab}	43.7±1.0^{b}
Alcalase + Thermoase	76.9±0.9^{ab}	87.9±0.2^{cd}	21.2±1.6^{ab}	55.2±0.2^{d}
Protex 6L + Protease S	75.1±0.5^{bc}	88.7±0.1^{b}	19.7±1.5^{bc}	45.1±0.3^{b}
Protex 6L + Thermoase	77.8±0.8^{a}	90.0±0.3^{a}	18.8±0.7^{c}	54.4±2.4^{d}
Protease S + Thermoase	69.4±1.4^{d}	88.8±0.2^{b}	16.9±0.4^{d}	52.7±3.4^{d}
Pepsin + Protex 50FP	68.8±0.6^{de}	88.1±0.01^{c}	12.8±0.1^{e}	50.3±3.3^{c}
Pepsin + Protex 26L	66.7±1.1^{e}	87.0±0.7^{e}	14.4±0.4^{e}	54.3±0.8^{d}
Protex 50FP + Protex 26L	74.0±0.3^{c}	88.0±0.4^{c}	20.6±0.4^{abc}	52.6±1.1 ^{d}

Working parameters are presented in Table S2. *ACE inhibition was determined at 0.05 mg/mL of the hydrolysate. Same superscripts in a column indicate no difference ($P < 0.05$).

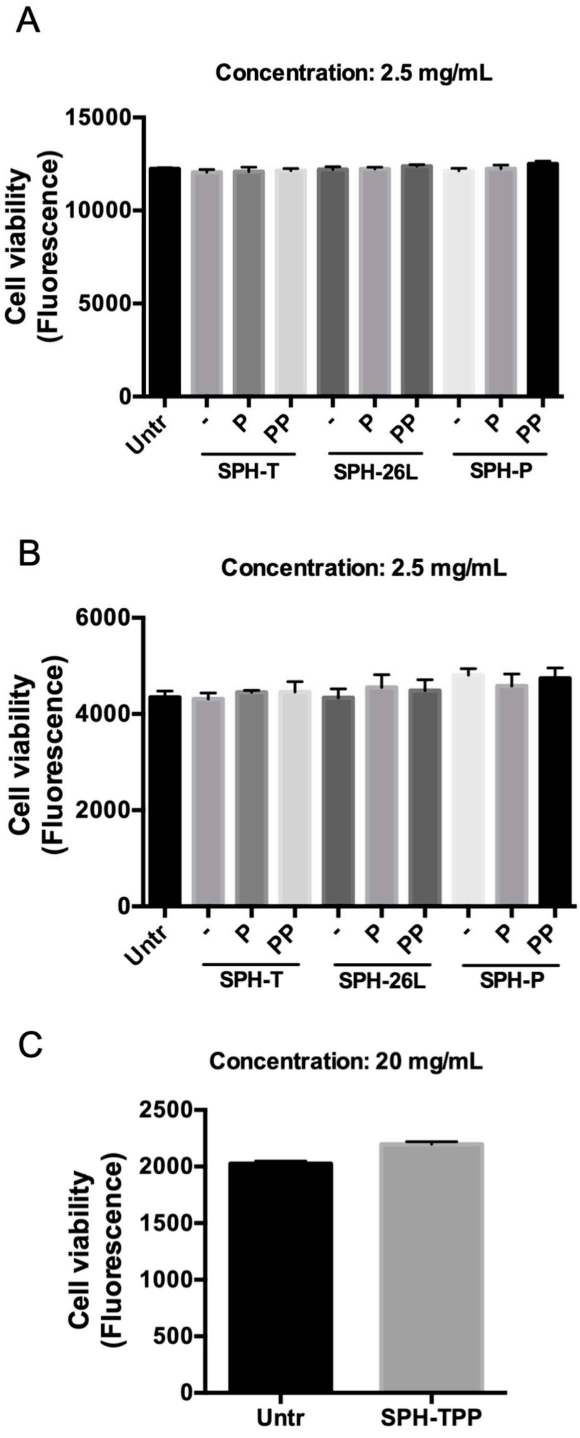

Figure S1 Cytotoxicity of SPHs in A7r5 (A), EA.hy 926 (B), and Caco-2 (C) cells (n = 6). -, P, and PP indicates non-, pepsin-, and (pepsin + pancreatin)-digestion, respectively. SPHs were tested at 2.5 mg/mL in A7r5 and EA.hy926 cells and 20 mg/mL in Caco-2 cells. All samples were treated for 24 hours, followed by alamarBlue® cell viability assay protocol provided by Thermo Fisher Scientific (Burlington, ON, Canada).

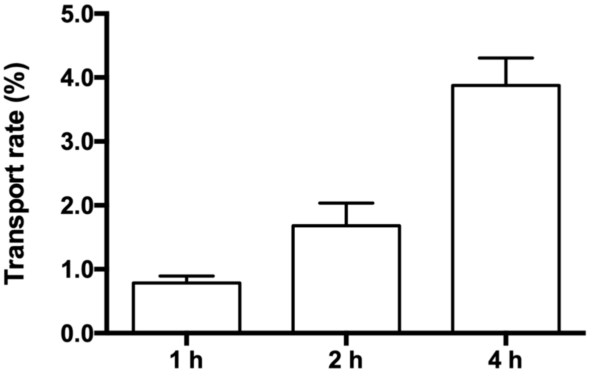

Figure S2 Transport rate of SPH-TPP across Caco-2 monolayers up to 4 h (n=6). Transport rate was calculated based on the % of peptides transported from the apical to the basolateral side

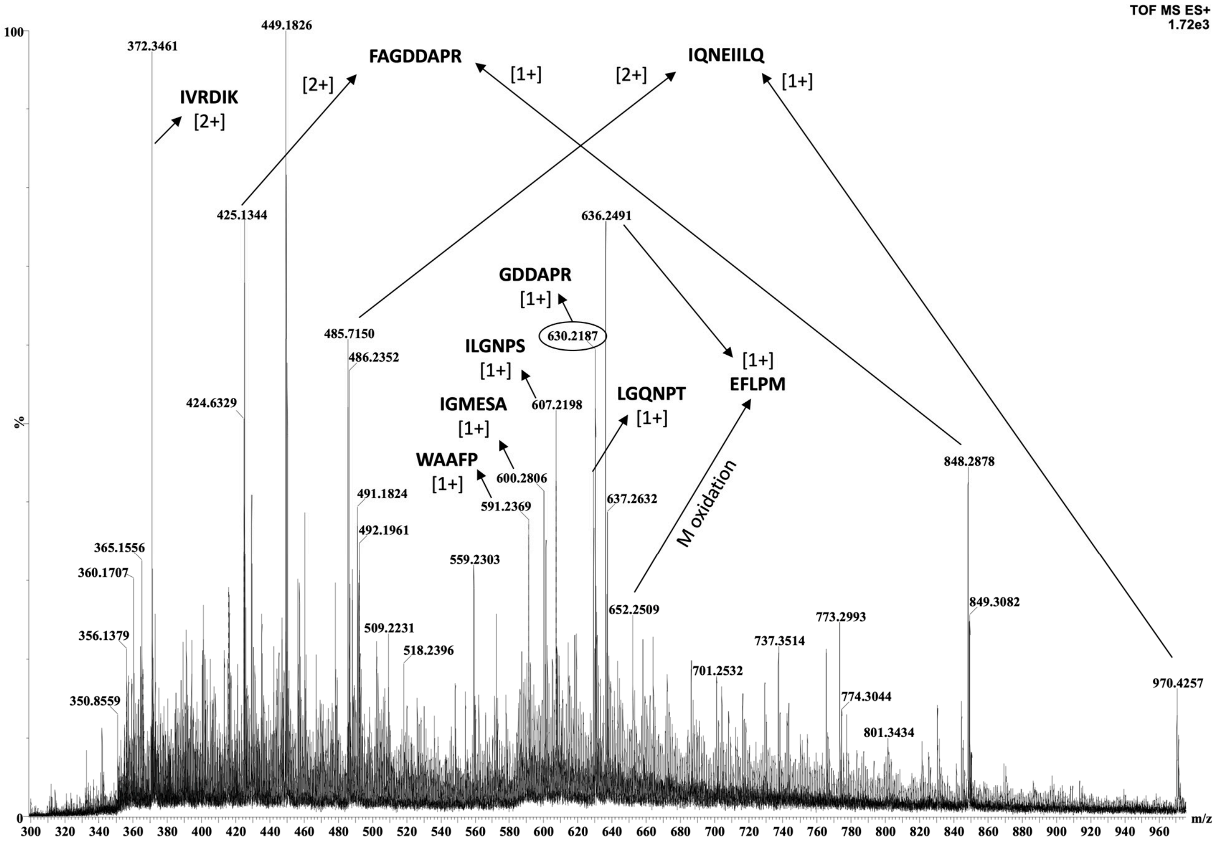

Peptides	Source	Observed m/z	Calculated mass
**IVRDIK**	Actin	372.35	742.47
**FAGDDAPR**	Actin	424.71 (848.29)	847.38
**IQNEIILQ**	Collagen α1 chain	485.72 (970.43)	969.48
**WAAFP**	not known	591.24	590.29
**ILGNPS**	Myosin light chain	600.28	599.33
**IGMESA**	Actin	607.22	606.27
**LGQNPT**	Myosin light chain	629.32	628.32
**GDDAPR**	Actin	630.22	629.28
**EFLPM**	myosin light chain	636.33	635.3

Figure S3 Mass spectrum of the major Caco-2 permeate of SPH-TPP at 4 h. The inserted table lists parent proteins and molecular weight of the identified peptides
